# The Effect of Caffeine on Wnt/β‐Catenine and P38 Mitogen‐Activated Protein Kinases (MAPK) Signal Pathways and Some Biochemical Parameters on Cafeteria Diet in Rats

**DOI:** 10.1002/fsn3.71138

**Published:** 2025-11-05

**Authors:** Lale Baser, Emine Atakisi

**Affiliations:** ^1^ Department of Biochemistry Kafkas University Faculty of Veterinary Medicine Kars Turkey

**Keywords:** β‐catenin, cafeteria diet, caffeine, MAPK, obesity, signaling pathways

## Abstract

Obesity is a major noncommunicable public health problem that is rapidly spreading worldwide, arising from an imbalance between energy intake and expenditure, and various interventions have been attempted for its treatment. This study evaluated the impact of caffeine on metabolic and hepatic parameters in rats with obesity induced by a cafeteria diet. Rats were assigned to control, caffeine, obesity, and obesity + caffeine groups. The cafeteria diet effectively promoted obesity, as evidenced by increased body weight, BMI, and Lee Index, accompanied by elevated serum glucose, triglycerides, total cholesterol, low‐density lipoprotein (LDL), very low‐density lipoprotein (VLDL), and reduced high‐density lipoprotein (HDL) levels. Obesity also led to higher plasma asprosin and visfatin levels, decreased hepatic β‐catenin and P38 mitogen‐activated protein kinases (p38 MAPK) expression, and histopathological alterations in liver tissue. Caffeine administration mitigated body weight gain, improved lipid profiles, and modulated plasma levels of asprosin, preptin, and visfatin, while reducing liver aspartate aminotransferase (AST) and alanine aminotransferase (ALT) activities. Although caffeine did not restore β‐catenin or p38 MAPK protein levels in obese rats, it alleviated liver histopathological damage. These findings indicate that caffeine may exert protective effects against cafeteria diet‐induced obesity by regulating multiple metabolic parameters and improving liver morphology. The study highlights the potential of caffeine as a modulator of obesity‐related metabolic dysregulation, while suggesting that further research is necessary to clarify its influence on β‐catenin and p38 MAPK signaling pathways.

## Graphical abstract

In this article, the effect of the cafeteria diet on obesity formation and the effect of caffeine on obesity recovery are explained through various signaling pathways and hormones.

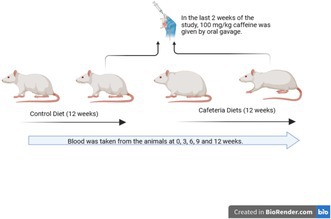

## 1 Introduction

The prevalence of overweight and obesity is increasing worldwide and has nearly doubled since 1980. Currently, almost one‐third of the global population is classified as either overweight or obese (Chooi et al. 2019). Obesity, which has become a major public health issue, not only disrupts nearly all physiological processes in the body but also contributes to the development of metabolic diseases (such as diabetes mellitus and fatty liver), cardiovascular disorders, musculoskeletal conditions, neurological disorders including Alzheimer's disease, and various types of cancer (Blüher 2019; Chooi et al. 2019). These conditions have been reported to lead to a decline in quality of life and work productivity (Chooi et al. 2019).

Regardless of geographic location, ethnicity, or socioeconomic status, the primary factor in the development of obesity at any age has been reported to be an imbalance between calories consumed and calories expended (Blüher 2019; Chooi et al. 2019). In addition to nonmodifiable factors such as genetic predisposition and hypothalamic abnormalities, modifiable factors also play a significant role in the development of obesity. These include epigenetic regulation, environmental conditions, dietary habits, physical activity levels, sleep duration, medication use, socioeconomic status, psychosocial stress, exposure to endocrine disruptors, and variations in gut microbiota (Masood and Moorthy 2023).

Caffeine, also known as 1,3,7‐trimethylxanthine, is a naturally occurring purine alkaloid that is widely consumed worldwide and possesses highly beneficial pharmacological effects. In the liver, caffeine is metabolized by cytochrome P450 1A2 (CYP1A2) into paraxanthine, theobromine, and theophylline. Caffeine increases energy expenditure and fat tissue oxidation by stimulating the sympathetic nervous system, thereby reducing weight gain and fat tissue ratio. Thus, when used in combination with other treatments, it provides highly successful outcomes in the treatment and prevention of obesity (Zhao et al. 2023).

Caffeine exerts anti‐obesity effects through multiple biological mechanisms. It suppresses key regulators of adipogenesis and inhibits proteins involved in lipid synthesis, leading to the formation of smaller lipid droplets in adipocytes (Fortunato et al. 2024; Yinghao et al. 2025). In addition, caffeine stimulates the breakdown of triglycerides (lipolysis), partially activates hormone‐sensitive lipase, and enhances thermogenesis (heat production) by upregulating pathways such as PPARα and PGC1α, which promote fatty acid oxidation and brown adipose tissue activity. Finally, its ability to modulate gut microbiota and their metabolites contributes to the regulation of host metabolism and the reduction of fat deposition (Fortunato et al. 2024).

Asprosin is an adipokine produced through the cleavage of fibrillin‐1, which acts via the OLFR734/OR4M1 receptor in the liver to stimulate glucose production and increase appetite. Clinical studies have reported elevated asprosin levels in metabolic disorders such as obesity, type 2 diabetes, and non‐alcoholic fatty liver disease, indicating that asprosin is associated with key metabolic parameters (Shabir et al. 2021). Adropin, encoded by the ENHO gene, is primarily produced in the liver and brain, but is also expressed in the heart, kidneys, and other organs. Circulating adropin exerts multifaceted effects in the body, including enhancing glucose utilization, improving insulin resistance, regulating lipid metabolism, supporting cardiovascular function, and modulating inflammation (Ali et al. 2022; Jasaszwili et al. 2020). Visfatin, classified as an adipokine and also known as nicotinamide phosphoribosyltransferase (NAMPT), is primarily synthesized by visceral adipose tissue. It has been reported to influence plasma glucose levels by mimicking insulin's effects (Fukuhara et al. 2005). Visfatin plays a significant role in obesity‐associated metabolic and inflammatory disorders, and most studies have reported elevated plasma or tissue levels in obese rats (Nanda et al. 2024; Sun et al. 2012). Preptin, a 34‐amino acid peptide primarily secreted by pancreatic beta cells, plays a role in glucose‐mediated insulin secretion. Preptin levels have been associated with various metabolic conditions, including diabetes, polycystic ovary syndrome (PCOS), gestational diabetes, and osteoporosis (Aydin 2014).

The cafeteria diet model is established by supplementing standard rat chow with high‐calorie processed foods and unhealthy snacks readily available from grocery stores (Tseng et al. 2024). It consists of packaged foods that are easily accessible to people, such as cookies, cakes, processed meats, and potato chips, which are quite tasty and diverse, high in energy and low in nutritional value. This dietary regimen induces obesity, metabolic syndrome, and hyperphagic behavior in rodents, thereby mirroring the pathophysiological consequences of junk food consumption in humans, and is regarded as a highly reliable model for the induction of obesity (Lalanza and Snoeren 2021). Compared to traditional high‐fat diets, the cafeteria diet has been reported in the literature to more accurately and realistically reflect obesity development in experimental animals. In models of obesity induced by this diet, animals exhibit obesity phenotypes characterized by hyperphagia, increased body fat and weight gain, elevated blood glucose levels, insulin resistance, and various alterations in blood lipid profiles (Tseng et al. 2024). It has been demonstrated that the cafeteria diet alters feeding patterns in rodents, reduces the hedonic value of alternative rewards, and tends to impair stress regulation and spatial memory. However, its effects on impulsivity, coping mechanisms, and social behavior remain to be fully elucidated. One of the main limitations of the cafeteria diet model is the variability in the nutritional content of the foods used, making it challenging to control caloric density. This variability can negatively impact the reproducibility and standardization of the model. Additionally, the high cost of implementing the cafeteria diet model and differences in its application across laboratories are significant factors that limit its widespread use (Lalanza and Snoeren 2021).

Wnt signaling is a growth control pathway involved in the development of the organism, regulation of tissues, and cell proliferation (Liu et al. 2022). The Wnt signaling pathway is reported to play important roles in obesity. It has been reported that the β‐catenin–dependent branch of Wnt is involved in restricting the development of adipocytes, while the non–β‐catenin‐dependent branch is involved in promoting adipogenesis with Wnt5a and Wnt5b (Aamir et al. 2020). The Wnt/β‐catenin signaling pathway prevents obesity by inhibiting C/EBPα and PPARγ signaling pathways. It can also inhibit adipogenesis and differentiation of preadipocytes (Luo et al. 2022).

The mitogen‐activated protein kinase (MAPK) signaling pathway is a signal transduction pathway involved in the regulation of numerous cellular processes such as growth, proliferation, and differentiation of cells in response to various stimuli from outside the cell, response to stress, cell migration, and apaptosis (Fan et al. 2019). Although metabolic stress factors such as inflammation and obesity have been reported to cause activation of p38 MAPK and JNK signaling pathways, the results of some studies report the opposite. This reflects the individual effects of MAPKs in metabolism (Solinas and Becattini 2017).

In this study, the effect of caffeine on cafeteria diet‐induced obesity was investigated. Body weight, BMI, Lee Index, serum glucose, blood lipid profile, AST and ALT activities, and plasma levels of asprosin, adropin, preptin, and visfatin were evaluated; moreover, alterations in hepatic Wnt/β‐catenin and p38 MAPK signaling pathways were examined, and histopathological analysis of liver tissue was performed.

## 2 Materials and Methods

### 2.1 Chemicals

Pure caffeine obtained from Merk (Sigma‐Aldrich, CAS Number: 58‐08‐2, Merck KGaA, Darmstadt, Germany) (Sigma‐Aldrich brand) was dissolved in saline and used in the experimental phase. The chemicals used for Western blot analysis, including NaCl (CAS No: 7647‐14‐5), Na_2_HPO_4_·12H_2_O (CAS No: 10039‐32‐4), NaH_2_PO_4_·12H_2_O (CAS No: 10049‐21‐5), NP‐40 (CAS No: 9016‐45‐9), Tris–HCl (CAS No: 1185‐53‐1), Aprotinin (CAS No: 9087‐70‐1), Tris base (CAS No: 77‐86‐1), Sodium dodecyl sulfate (CAS No: 151‐21‐3), and Tween 20 (CAS No: 9005‐64‐5), were obtained from Merck KGaA (Darmstadt, Germany) under the Sigma‐Aldrich brand, Methanol (CAS No: 67‐56‐1, Isolab by Interlab, Turkey) Bradford Reagent (CAS No: 5000205, 5000205EDU, Bio‐Rad Laboratories Inc., Turkey) 4× Laemmli (CAS No: 1610747, Bio‐Rad Laboratories Inc., Turkey).

### 2.2 Animals and Experimental Design

All experimental animals were obtained from Atatürk University Medical Experimental Application and Research Center and transferred to Kafkas University Experimental Animals Research and Application Center. A total of 37 male Wistar rats weighing 250–300 g (10–12 weeks old) were used in the study. The experimental animals were kept in special cages without any contact between the groups. During the 10‐day adaptation period, all experimental animals were fed *ad libitum* with standard rat chow and drinking water was added to their drinkers daily. Rats were housed in plastic cages with sawdust litter with 5 rats in each cage during the whole experimental period. The litter was cleaned regularly every week. They were housed in rooms with 12 h light/12 h dark lighting and 22°C ± 2°C temperature. Body weights of the rats in all groups were recorded every day using a digital weighing scale.

Experimental animals were divided into four groups as control (group I), caffeine (group II), obesity (group III), and obesity + caffeine (Group IV): **Group I (control group, *n* = 8):** Fed with standard rat chow for 12 weeks and no treatment was applied. **Group II (caffeine group, *n* = 10):** Fed with standard rat chow for 12 weeks and 100 mg/kg caffeine was administered by oral gavage for 14 days starting from week 10. **Group III (obesity group, *n* = 10):** Fed with cafeteria diet for 12 weeks and no other treatment was applied. **Group IV (obesity + caffeine group, *n* = 9):** They were fed a cafeteria diet for 12 weeks and 100 mg/kg caffeine was administered by oral gavage for 14 days starting from week 10.

### 2.3 Ethics Committee Approval

The necessary approval for the study was obtained from Kafkas University Experimental Animals Local Ethics Committee (KAU‐HADYEK) with the protocol number KAU‐HADYEK/2021‐121.

### 2.4 Cafeteria Diet

The cafeteria diet provided for feeding the rats in the obesity and obesity + caffeine groups for 12 weeks during the experiment was prepared by Arden Araştırma Deneysel Tıp Malzemeleri Experimental Animal Feed Trade. The cafeteria diet was prepared in pellet form and provided to the rats. The composition of the diet is shown in Table 1.

**TABLE 1:** Analysis results of the feed used in the cafeteria diet.

Control diet (standard rat feed)			Cafeteria diet	
Parameter	Analysis result %	Calories (kcal)	Analysis result (%)	Calories (kcal)
Moisture	—	—	7.65	—
Dry matter	—	—	92.35	37.2
Protein	23.90	95.60	9.25	109.1
Fat	5	45	12.12	225.4
Carbohydrate	48.70	194.80	56.36	206.48

### 2.5 Determination of Obesity

#### 2.5.1 Measurement of Body Weight and Height

Obesity formation was determined by calculating body mass index (BMI) and Lee Index every week (Novelli et al. 2007). Body weights of the animals were weighed every week with a scale. The height of the animals was recorded by measuring the distance between the nose and anus with a thin, inflexible tape measure (Novelli et al. 2007). The following formula was used to calculate the BMI value: BMI = g/cm^2^. The range of 0.45–0.68 g/cm^2^ was considered a normal range, while rats with BMI values greater than 0.68 g/cm^2^ were considered obese (Novelli et al. 2007).

The following formula was used to calculate the Lee index: Rats with a Lee Index greater than 0.3 g/cm were considered obese (Lee Index = Cube root of body weight (g) ÷ Nose‐to‐anus length (cm)) (Lee 1929). In experimental animals, the Lee index is calculated by dividing the cube root of body weight by the naso‐anal length (Bernardis and Patterson 1968; Macêdo et al. 2021; Malafaia et al. 2013; Rogers and Webb 1980). It serves as a rapid and non‐invasive indicator for estimating obesity and body fat; however, its advantage over other measurements such as BMI or fat pad weight is modest, and it provides more accurate results when used in combination with other parameters (Macêdo et al. 2021; Malafaia et al. 2013; Rogers and Webb 1980).

### 2.6 Blood Collection From Live Animals

Blood was collected from the submandibular vein of all rats at weeks 0, 3, 6, 9, and 12 and transferred into sterile Eppendorf tubes. Samples were centrifuged at 3000 rpm for 10 min at +4°C, and the serum was separated and transferred into labeled sterile Eppendorf tubes. The serum samples were stored at −20°C for biochemical analyses. Blood sampling from live animals was performed to assess the effects of obesity development and caffeine administration on serum lipids.

### 2.7 Sample Collection and Preparation

At the end of the experiment, overnight fasted rats were anesthetized with 50 mg/kg ketamine and 10 mg/kg xylazine. The rats were euthanized and liver tissues were removed from each rat. They were washed with phosphate‐buffered saline (PBS) and then divided into two parts: one piece was homogenized in its own lysis buffer for further Western Blotting, in ice‐cold PBS for Autoanalyzer and ELISA testing and the other piece was fixed in phosphate‐buffered formalin for histological examination with hematoxylin and eosin.

### 2.8 Biochemical Analyses

#### 2.8.1 Hormones Involved in Lipid Metabolism

Analysis of asprosine, adropin, preptin, and visfatin in plasma samples from rats was performed by spectrophotometer using a commercial ELISA kit (BT LAB, Shanghai Korain Biotech Co. Ltd., China).

#### 2.8.2 Blood Glucose

Blood glucose levels in serum samples obtained from rats at the end of 0, 3, 6, 9, and 12 weeks were determined using an autoanalyzer (Beckman Coulter, AU5800, Brea, California, USA).

#### 2.8.3 Blood Lipids

Total cholesterol and triglyceride levels in blood samples obtained from rats at weeks 0, 3, 6, 9, and 12 were determined using an autoanalyzer (Beckman Coulter, AU5800, Brea, California, USA), and VLDL levels were calculated using the formula Triglyceride/5. HDL and LDL levels were measured from serum samples separated from blood collected at week 12.

#### 2.8.4 Liver Function Enzymes

AST and ALT enzyme activities in serum samples obtained from rats at the end of 0, 3, 6, 9, and 12 weeks were determined using an autoanalyzer (Beckman Coulter, AU5800, AU5800, Brea, California, USA).

### 2.9 Western Blot

Total protein from liver tissues was homogenized using cold NP‐40 lysis buffer containing 10 mg/mL aprotinin. Protein concentrations were measured by the Bradford method by recording absorbance at 595 nm using bovine serum albumin (BSA) as the standard (Bradford 1976). Standards in the range 2–12 μg/mL were prepared from a 0.2 mg/mL BSA stock solution. After incubation for 10 min at room temperature with the addition of Bradford reagent, protein concentrations of tissue samples were measured at 595 nm according to the BSA standard curve. Sodium dodecyl sulfate‐polyacrylamide gel electrophoresis (SDS‐PAGE) was performed according to Laemmli (1970). Protein samples were loaded on the SDS‐PAGE gel (10%), electrophoretically separated, and transferred to a nitrocellulose membrane. Membranes were blocked with 5% skim milk powder for 1 h at room temperature. Immunoblots were incubated overnight at 4°C with a primary antibody against p38 MAPK (sc: 7972, Santa Cruz Biotechnology Inc. Santa Cruz Biotechnology Inc. Dallas, Texas, USA) (1:1000) and β‐catenin (sc:7963, Santa Cruz Biotechnology Inc. Santa Cruz Biotechnology Inc. Dallas, Texas, USA) (1:1000). After washing with tris‐buffered saline containing Tween 20, antibody binding was detected using HRP‐conjugated secondary antibody (sc:2357, Santa Cruz Biotechnology Inc. Santa Cruz Biotechnology Inc. Dallas, Texas, USA) (1:2000 dilution) for 1 h and ECL Western blot substrate. Immunoblots were visualized using a chemiluminescence imaging system (iBrightCL 1000, Invitrogen, California, USA) and quantified using Image J software. Normalized with β‐actin (sc:47778, Santa Cruz Biotechnology Inc., Dallas, Texas, USA) (1:1000) as an internal control. To control for sampling errors, the ratio of band intensities to β‐actin was obtained to quantify the relative protein expression level.

### 2.10 Histopathologic Evaluation

Hematoxylin–eosin staining was performed to determine the pathological changes in the liver. The preparations were examined under a light microscope (Olympus Bx53) and photographed using the Cell ^P program (Olympus Soft Imaging Solutions GmbH, 3,4).

### 2.11 Statistical Analyses

GraphPad Prism 8.0.2 program was used for statistical analysis of the data obtained from the studies. Depending on whether the data were normally distributed, either parametric or nonparametric ANOVA was performed. Differences between groups in the analysis of body weight, BMI, Lee Index, glucose, total cholesterol, triglycerides, VLDL‐cholesterol, AST, and ALT data were determined using the two‐way ANOVA (Tukey) test. LDL, HDL‐cholesterol, asprosin, adropin, preptin, visfatin, β‐catenin, and p38 MAPK data were analyzed by one‐way analysis of variance (one‐way ANOVA), and differences between groups were determined by the Duncan test. *p* < 0.05 level was considered statistically significant. For Western blotting, the intensity of the bands was determined by ImageJ software (National Institutes of Health, Bethesda, MD, USA) and normalized with β‐actin (1:1000) as an internal control.

## 3 Results

### 3.1 Live Weight Change

**FIGURE 1:**
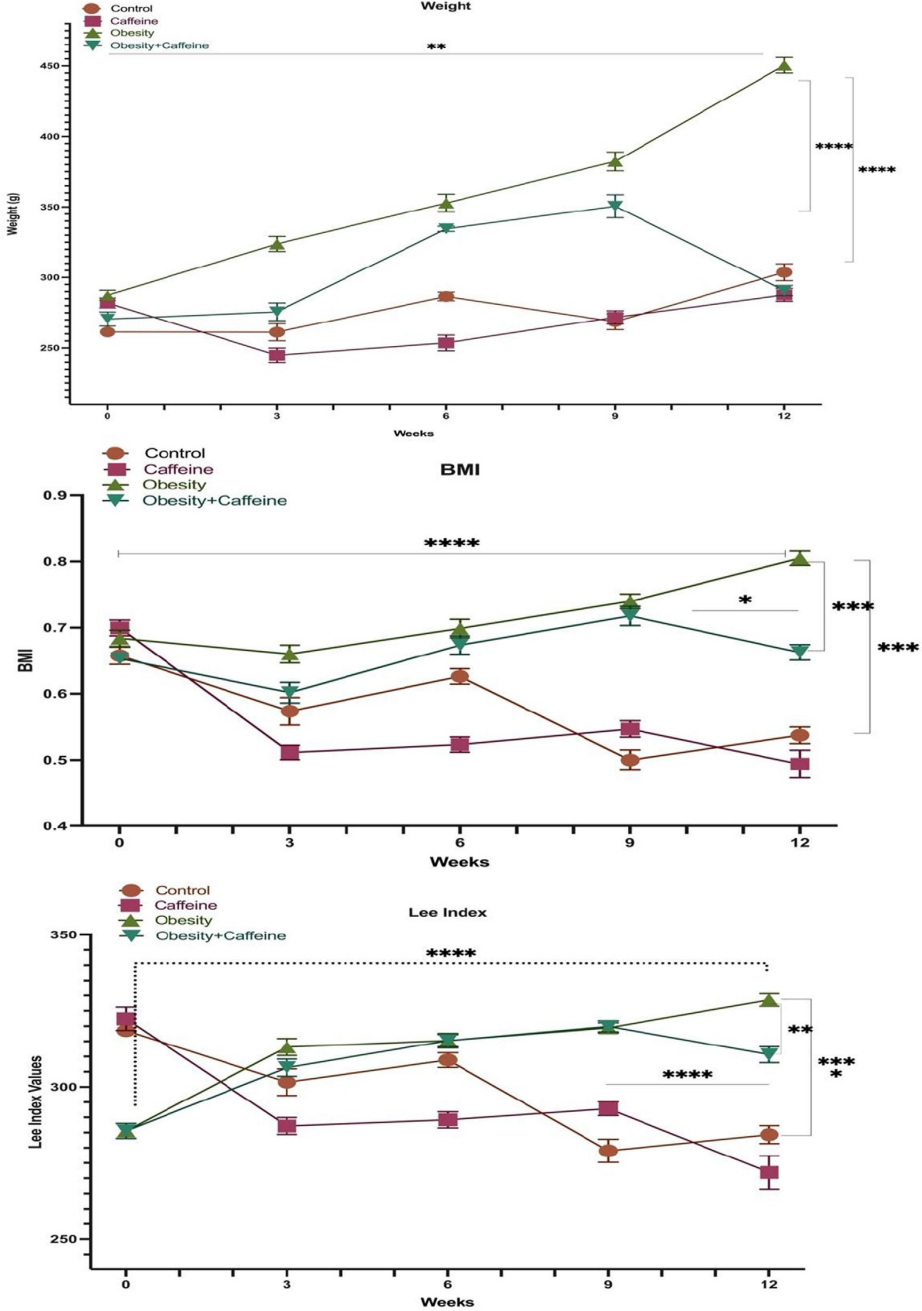
Average body weight (g), BMI, and Lee Index changes of the groups during the experiment. The bars with vertical lines represent standard deviations, while the asterisks indicate statistically significant differences between groups (*p* < 0.05). The asterisks indicating statistical differences between groups represent the following: *p < 0.05, **p < 0.01, ***p < 0.001, and ****p < 0.0001. The study included the following sample sizes: control group (*n* = 8), caffeine group (*n* = 10), obesity group (*n* = 10), and obesity + caffeine group (*n* = 9). Error bars represent the standard error of the mean (SEM).

Live weight changes of the groups are shown in Figure 1. When the live end weights were analyzed, it was observed that the live weights increased statistically significantly (*p* < 0.001) when the obesity group was compared to the control group, and the statistical difference (*p* < 0.001) between the control group and obesity + caffeine group at week 10 was closed at week 12. The difference between weeks 0 and 12 in the obesity group (*p* < 0.001) shows the effect of a cafeteria diet on body weight, and the difference between the obesity group and obesity + caffeine group at week 12 (*p* < 0.001) shows the effect of caffeine on body weight.

Obesity formation in rats on a cafeteria diet and the effect of caffeine on obesity were investigated. To determine obesity formation, height and weight values were measured every week and BMI values were calculated and given in Figure 1. When BMI values were analyzed, it was observed that there was a statistically significant difference (*p* < 0.001) between the obesity group and the control group. The difference between weeks 0 and 12 in the obesity group (*p* < 0.01) shows the effect of the cafeteria diet on BMI values; the difference between the obesity group and the obesity + caffeine group at week 12 (*p* < 0.001) and the statistical difference between weeks 10 and 12 in the obesity + caffeine group (*p* < 0.05) and between weeks 9 and 12 in the caffeine group (*p* < 0.001) shows the effect of caffeine on BMI.

Another index for the evaluation of obesity formation in rats is the Lee Index. The Lee Index was calculated by measuring height and weight every week to examine the formation of obesity in rats and the effect of caffeine on obesity and these data are given in Figure 1. When Lee Index values were analyzed, it was observed that there was a statistically significant difference (*p* < 0.001) between the obesity group and the control group. The difference between weeks 0 and 12 in the obesity group (*p* < 0.001) shows the effect of the cafeteria diet on Lee Index values; the difference between the obesity group and the obesity + caffeine group at week 12 (*p* < 0.01) and the statistical difference between weeks 9 and 12 in the caffeine group (*p* < 0.05) show the effect of caffeine on Lee Index.

### 3.2 Western Blot Analysis Results

**FIGURE 2:**
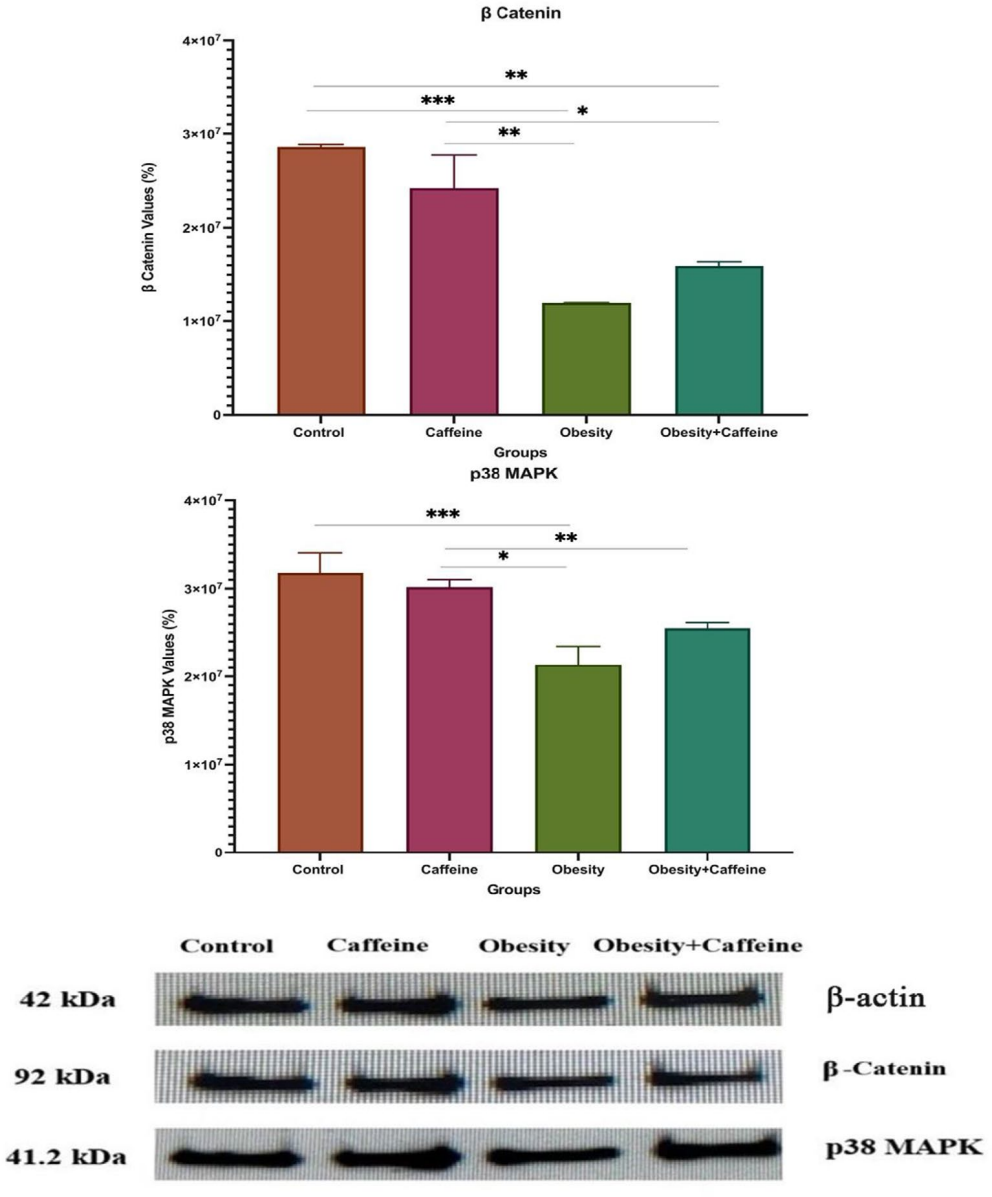
Band intensities of proteins in liver tissues and liver β‐catenin and p38 MAPK expression levels. The bars with vertical lines represent standard deviations, while the asterisks indicate statistically significant differences between groups (*p* < 0.05). The asterisks indicating statistical differences between groups represent the following: *p < 0.05, **p < 0.01, and ***p < 0.001. The study included the following sample sizes: control group (*n* = 8), caffeine group (*n* = 10), obesity group (*n* = 10), and obesity + caffeine group (*n* = 9). Error bars represent the standard error of the mean (SEM).

The band changes of β‐actin, β‐catenin, and p38 MAPK proteins, whose levels were determined by Western blotting, in liver tissue are shown in Figure 2 and liver tissue expression levels are shown in Figure 2. The expressions of the proteins were standardized according to β‐actin expression and relative percentage values were calculated compared to the control.

As seen in Figure 2, when the expression levels of liver β‐catenin protein between the groups are compared with the other groups, there is a statistical difference between control and obesity (*p* < 0.001), obesity + caffeine, caffeine and obesity (*p* < 0.01) and the obesity + caffeine group (*p* < 0.05).

As seen in Figure 2, when the expression levels of p38 MAPK protein in the liver were compared with the other groups, there was a statistical difference between control and obesity (*p* < 0.001), caffeine and obesity (*p* < 0.05) and the obesity + caffeine group (*p* < 0.01).

### 3.3 Plasma Asprosin, Adropin, Preptin, and Visfatin Levels

**FIGURE 3:**
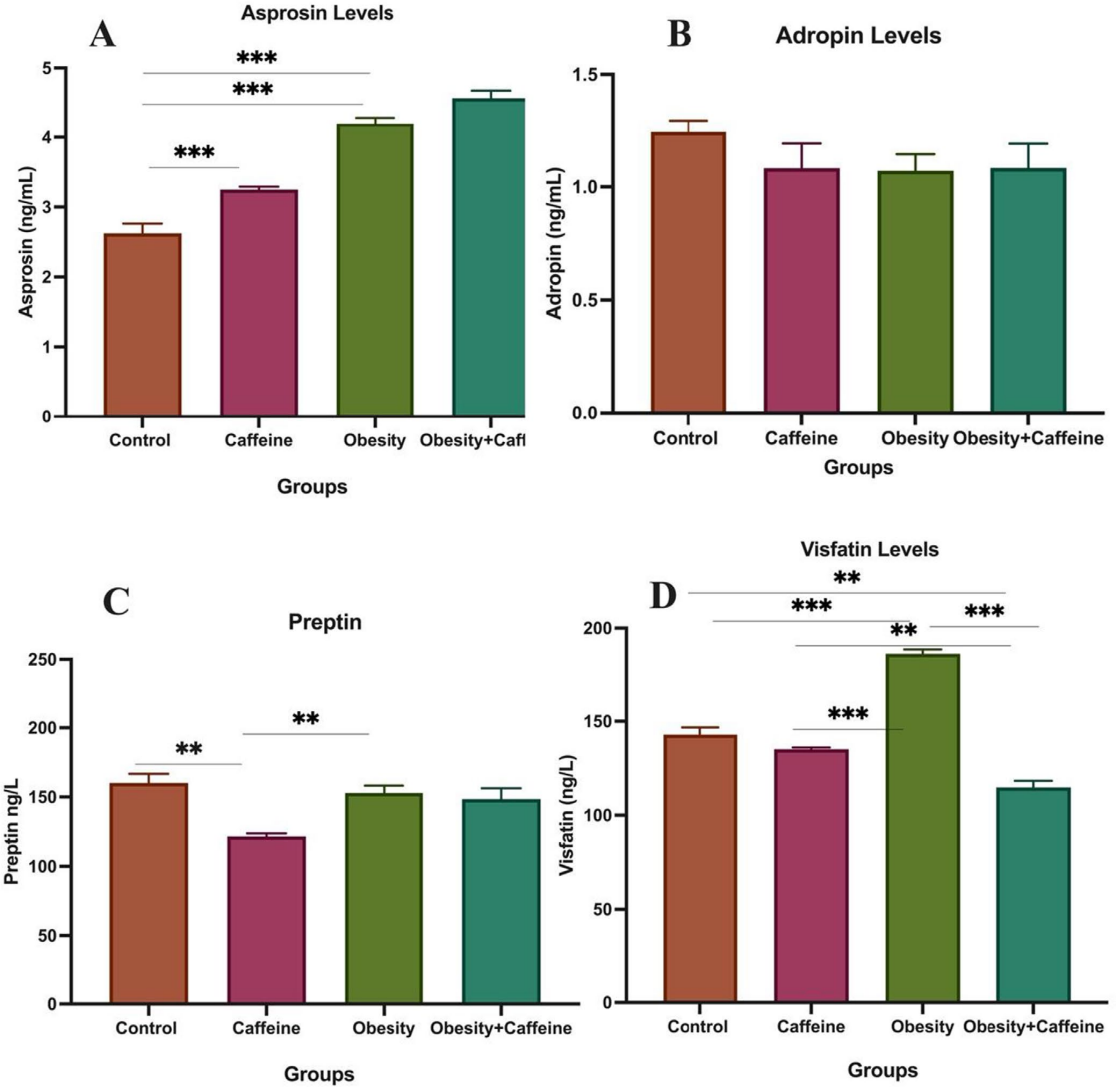
(A) Plasma asprosin levels, (B) plasma adropin levels, (C) plasma preptin levels, (D) plasma visfatin levels. The bars with vertical lines represent standard deviations, while the asterisks indicate statistically significant differences between groups (*p* < 0.05). The study included the following sample sizes: control group (*n* = 8), caffeine group (*n* = 10), obesity group (*n* = 10), and obesity + caffeine group (*n* = 9). The asterisks indicating statistical differences between groups represent the following: *p < 0.05, **p < 0.01, and ***p < 0.001. The study included the following sample sizes: control group (*n* = 8), caffeine group (*n* = 10), obesity group (*n* = 10), and obesity + caffeine group (*n* = 9). Error bars represent the standard error of the mean (SEM).

Plasma asprosine levels between the groups in the study are shown in Figure 3A. According to the results obtained from the study, it was determined that the highest asprosine levels were in the obesity + caffeine group and the lowest asprosine levels were in the control group. As a result of the statistical analysis, a difference was found only between the control group and obesity, obesity + caffeine, and caffeine groups (*p* < 0.001).

Plasma adropin levels between the groups are shown in Figure 3B. According to the results obtained from the study, the highest plasma adropin levels were found in the control group and the lowest plasma adropin levels were found in the obesity group. No difference was found between the groups as a result of statistical analysis.

Plasma preptin levels between the groups are shown in Figure 3C. According to the results obtained from the study, it was determined that the highest plasma preptin levels were in the control group and the lowest plasma preptin levels were in the caffeine group. As a result of the statistical analysis, a difference was found between the control group and caffeine group (*p* < 0.001) and between the caffeine and obesity group (*p* < 0.01).

Plasma visfatin levels between the groups are shown in Figure 3D. According to the results obtained from the study, it was determined that the highest plasma visfatin levels were in the obesity group and the lowest plasma visfatin levels were in the caffeine group. As a result of the statistical analysis, a difference was found between control and obesity + caffeine (*p* < 0.01) and obesity, caffeine, and obesity (*p* < 0.001) and obesity + caffeine (*p* < 0.01), obesity and the obesity + caffeine group (*p* < 0.001).

### 3.4 Serum Glucose, Total Cholesterol, Triglyceride and VLDL Levels, and LDL and HDL Levels

**FIGURE 4:**
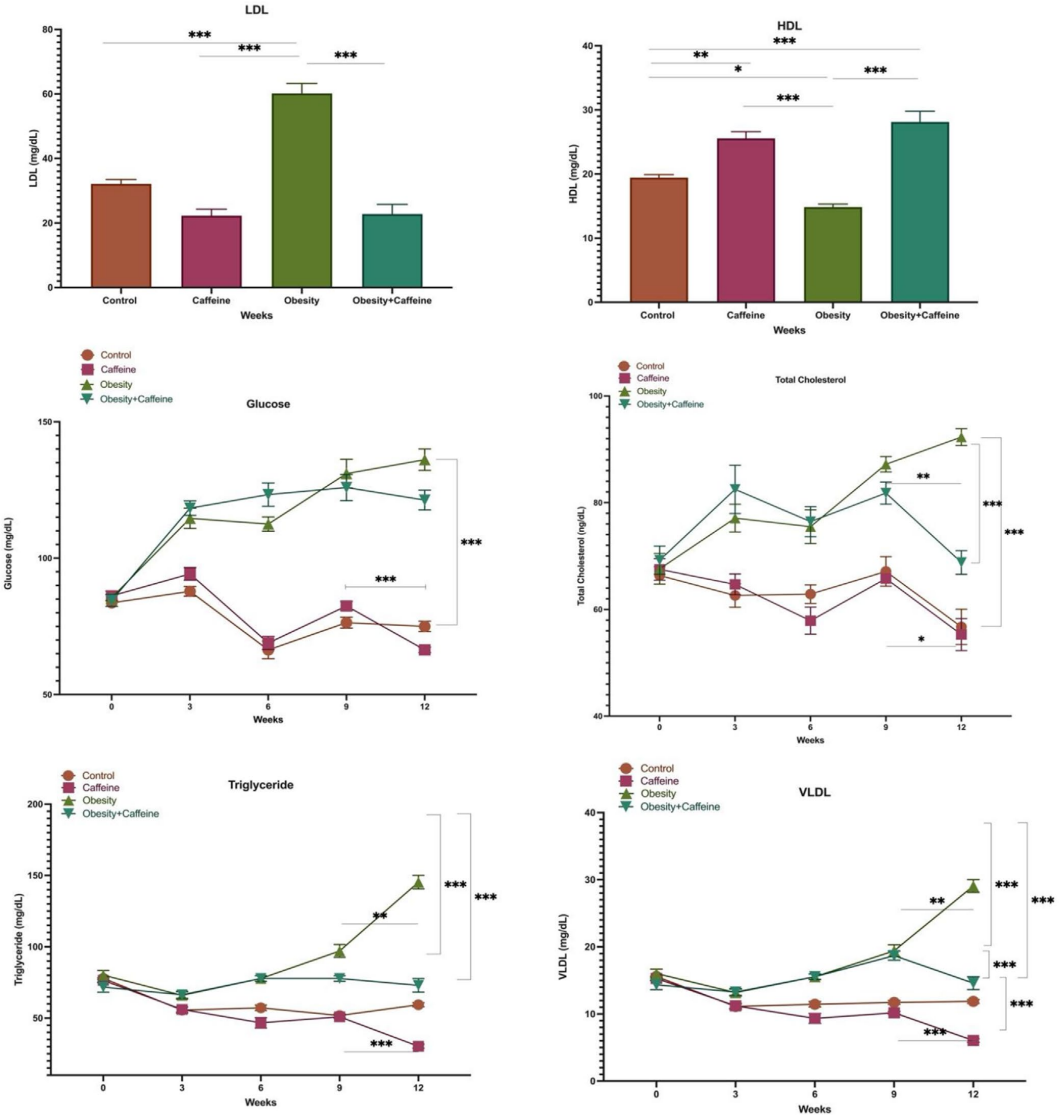
Serum HDL, LDL, glucose, total cholesterol, triglyceride, and VLDL levels. The bars with vertical lines represent standard deviations, while the asterisks indicate statistically significant differences between groups (*p* < 0.05). The asterisks indicating statistical differences between groups represent the following: *p < 0.05, **p < 0.01, and ***p < 0.001. The study included the following sample sizes: control group (*n* = 8), caffeine group (*n* = 10), obesity group (*n* = 10), and obesity + caffeine group (*n* = 9). Error bars represent the standard error of the mean (SEM).

Serum LDL and HDL‐cholesterol levels were measured by autoanalyzer at week 12. Statistical data of serum LDL are given in Figure 4. There is a statistical difference only between obesity and control, caffeine and obesity + caffeine (*p* < 0.001) groups. The difference between obesity and control group (*p* < 0.001) shows the effect of cafeteria diet on LDL levels and the difference between obesity and obesity + caffeine group (*p* < 0.001) shows the effect of caffeine on LDL levels. Serum HDL statistical data are also given in Figure 4. There is a statistical difference between control and obesity (*p* < 0.05), caffeine (*p* < 0.01), obesity + caffeine, obesity and caffeine and obesity + caffeine (*p* < 0.001) groups. The difference between obesity and control group (*p* < 0.05) shows the effect of cafeteria diet on HDL levels, the difference between control and caffeine group (*p* < 0.01) and the difference between obesity and obesity + caffeine group (*p* < 0.001) shows the effect of caffeine on HDL levels.

Serum glucose, total cholesterol, triglyceride and VLDL levels were measured by autoanalyzer at weeks 0, 3, 6, 9, and 12. Statistical data of serum glucose are given in Figure 4. The difference between weeks 0 and 12 in the obesity group (*p* < 0.001) shows the effect of cafeteria diet on glucose levels, and the statistical difference between weeks 9 and 12 in the caffeine group (*p* < 0.001) shows the effect of caffeine on glucose levels. The statistical data of serum total cholesterol are also given in Figure 4. When total cholesterol levels were analyzed, it was observed that there was a statistically significant difference (*p* < 0.001) between the obesity group and the control group. The difference between weeks 0 and 12 in the obesity group (*p* < 0.001) shows the effect of cafeteria diet on total cholesterol levels; the difference between the obesity group and the obesity + caffeine group at week 12 (*p* < 0.001), obesity + caffeine group (*p* < 0.01) and the statistical difference between weeks 9 and 12 in the caffeine group (*p* < 0.05) shows the effect of caffeine on total cholesterol levels.

Serum triglyceride and VLDL levels were measured by autoanalyzer at 0, 3, 6, 9, and 12 weeks. Statistical data of serum triglyceride levels are given in Figure 4. When triglyceride levels were analyzed, it was observed that there was a statistically significant difference (*p* < 0.001) between the obesity group and the control group. The difference between weeks 0 and 12 in the obesity group (*p* < 0.001) shows the effect of cafeteria diet on triglyceride levels; the difference between the obesity group and obesity + caffeine group at week 12 (*p* < 0.001), the obesity + caffeine group (*p* < 0.01) and the statistical difference between weeks 9 and 12 in the caffeine group (*p* < 0.001) show the effect of caffeine on triglyceride levels. Statistical data of serum VLDL levels are also given in Figure 4. When VLDL levels were analyzed, it was observed that there was a statistically significant difference (*p* < 0.001) between the obesity group and the control group. The difference between weeks 0 and 12 in the obesity group (*p* < 0.001) shows the effect of cafeteria diet on VLDL levels; the difference between the obesity group and obesity + caffeine group at week 12 (*p* < 0.001), the obesity + caffeine group (*p* < 0.01) and the statistical difference between weeks 9 and 12 in the caffeine group (*p* < 0.001) show the effect of caffeine on VLDL levels.

### 3.5 Serum AST and ALT Activities, Histopathologic Results, and Liver Weights

**FIGURE 5:**
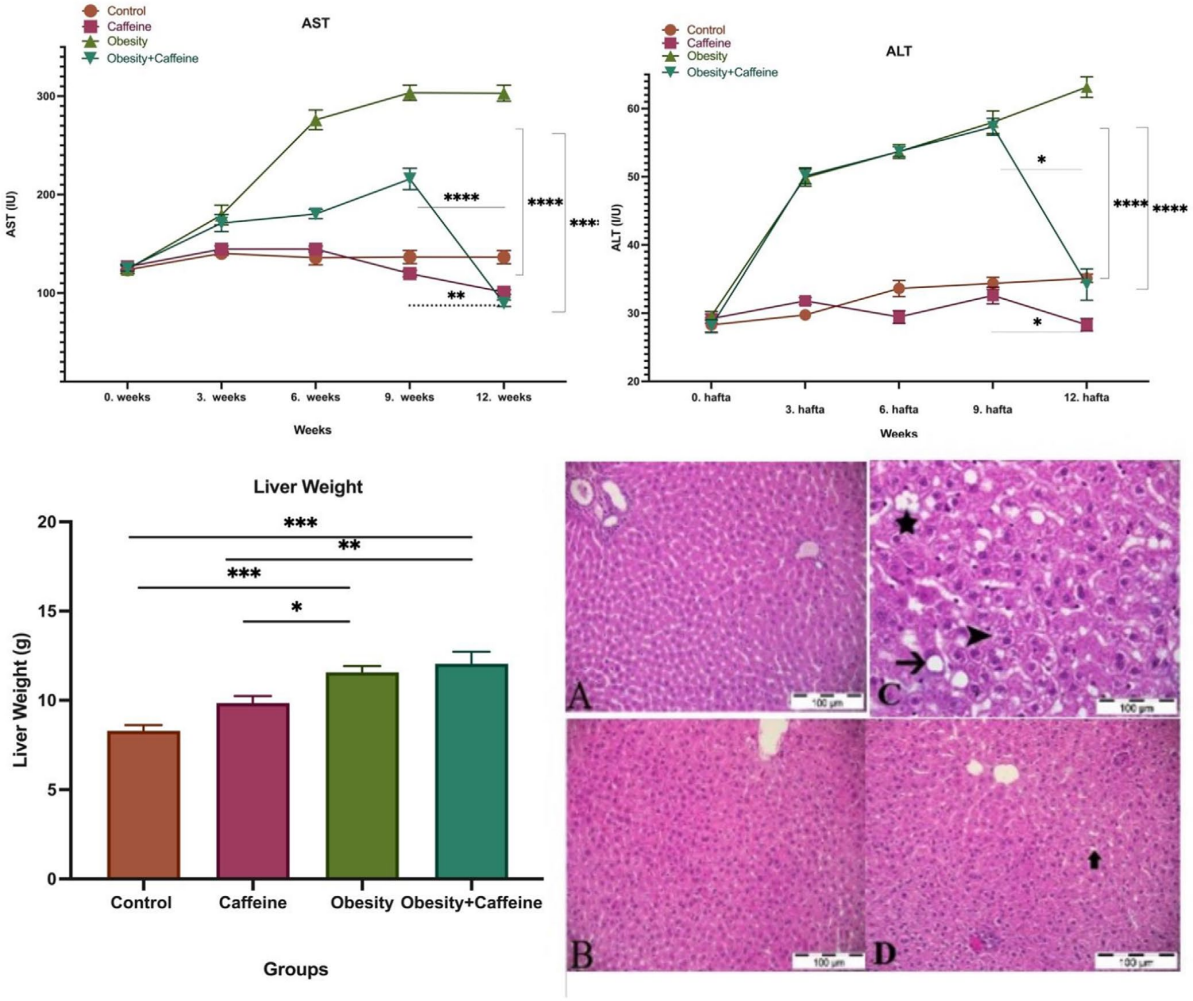
Serum ASLT, AST activities, liver weight, and histopathological images of liver tissue. The bars with vertical lines represent standard deviations, while the asterisks indicate statistically significant differences between groups (*p* < 0.05). (A) Control group, liver tissue; (B) caffeine group, liver tissue; (C) obesity group, hydropic degeneration in liver epithelial cells (arrowhead), fat degeneration (arrows) and fat vacuoles in hepatocyte cytoplasm (asterisk); (D) obesity + caffeine group, hydropic degeneration in liver epithelial cells (arrowheads), fat degeneration (arrows). Liver weights according to groups. The bars with vertical lines represent standard deviations, while the asterisks indicate statistically significant differences between groups (*p* < 0.05). The asterisks indicating statistical differences between groups represent the following: *p < 0.05, **p < 0.01, ***p < 0.001, and ****p < 0.0001. The study included the following sample sizes: control group (*n = 8*), *caffein*e group (*n* = 10), obesity group (*n* = 10), and obesity + caffeine group (*n* = 9). Error bars represent the standard error of the mean (SEM).

Serum AST and ALT activities were measured by autoanalyzer at weeks 0, 3, 6, 9, and 12. The statistical data of serum AST are given in Figure 5. When AST activities were analyzed, it was observed that there was a statistically significant difference (*p* < 0.001) between the obesity group and the control group. The difference between weeks 0 and 12 in the obesity group (*p* < 0.01) shows the effect of cafeteria diet on AST activity; the difference between obesity group and obesity + caffeine group at week 12 (*p* < 0.001), obesity + caffeine group (*p* < 0.001) and caffeine group between weeks 9 and 12 (*p* < 0.001) shows the effect of caffeine on AST activity. The statistical data of serum ALT are also given in Figure 5. When ALT activities were analyzed, it was observed that there was a statistically significant difference (*p* < 0.001) between the obesity group and the control group. The difference between weeks 0 and 12 in the obesity group (*p* < 0.001) shows the effect of cafeteria diet on ALT activity; the difference between obesity group and obesity + caffeine group at week 12 (*p* < 0.001), the difference between control and caffeine group (*p* < 0.05), obesity + caffeine group (*p* < 0.05) and the statistical difference between weeks 9 and 12 in caffeine group (*p* < 0.05) shows the effect of caffeine on ALT activity.

As a result of histopathological examinations, it was determined that the liver tissues from the control group exhibited a normal histological structure (Figure 5A). Similar to the control group, no pathologic changes were observed in the liver tissues of the animals in the second group, in which only caffeine was administered, and it was observed that the liver tissue maintained its normal histologic structure (Figure 5B). When obesity, control, and caffeine groups were compared, it was found that the liver epithelial cells of the animals underwent diffuse hydropic degeneration and single cell necrosis was observed. The boundaries of the degenerated cells were clear, the nucleus was in the center of the cell and the cytoplasm lost its ability to stain. In addition, fatty degeneration was observed in some hepatocytes. Large fat vacuoles were observed in the cytoplasm of degenerated cells and the nucleus was pushed aside (Figure 5C). In the obesity + caffeine group, compared to the obesity group, the damage was reduced, necrosis and hydropic degeneration were less and the lesions were mostly located in the periportal area (Figure 5D).

Liver weights between the groups are shown in Figure 5. According to the results obtained from the study, it was determined that the highest liver weight was in the obesity + caffeine group and the lowest liver weight was in the control group.

## 4 Discussion

When the mechanisms leading to obesity are examined, it is generally accepted that energy intake exceeds energy expenditure in the body. The excess energy is stored by adipocytes, making obesity inevitable. Obesity increases the risk of various diseases and conditions, thereby raising mortality rates. In addition to being highly costly for healthcare systems, it also imposes a financial burden on patients undergoing treatment (Lin and Li 2021). This study aimed to understand the process of obesity development in rats fed a cafeteria diet for 12 weeks and the effect of caffeine on this process. The findings indicate that the cafeteria diet significantly contributes to body weight gain, while caffeine helps reduce this weight gain. Analyzing the BMI and Lee Index data, it was concluded that the cafeteria diet was effective in inducing obesity, while caffeine successfully reversed the development of obesity, showing its potential in obesity treatment.

Although caffeine is widely consumed for its stimulant effects, it is also recognized as an influential factor in fat metabolism and weight management. Literature consistently indicates that caffeine can enhance fat oxidation, particularly during aerobic exercise; however, the magnitude of this effect depends on factors such as dose, timing of administration, individual fitness level, and habitual caffeine use. Caffeine stimulates the sympathetic nervous system, increasing catecholamine release, which in turn promotes lipolysis and elevates circulating free fatty acids to meet energy demands during exercise (Fernández‐Sánchez et al. 2024; Ramírez‐Maldonado et al. 2021). In previous studies, caffeine has been reported to have ameliorative effects on obesity and its associated complications in both rat and mouse models (de Souza et al. 2021; Zapata et al. 2020).

In obesity, appetite regulation is disrupted, and asprosin has been shown to increase appetite through the activation of AgRP neurons (Yuan et al. 2020). Most studies report that elevated circulating asprosin levels in obese adults, mice, and rats are pathologic (Duerrschmid et al. 2017; Mirr et al. 2023; Ozcan et al. 2022; Ugur et al. 2022; Ugur and Aydin 2019; Yuan et al. 2020). In the present study, which examined the effect of a cafeteria diet on plasma asprosin levels, it was concluded that the plasma asprosin levels of obese rats were higher than those of the control group. In the literature, only a very limited number of studies have investigated the effect of caffeine on asprosin release, and these studies are human studies, not experimental animal studies (Daneshyar 2024; Gkouskou et al. 2022).

In the present study, caffeine significantly increased plasma asprosin levels compared to the control group; however, no significant differences were observed between the obesity and obesity + caffeine groups. As an orexigenic hormone, the increase in asprosin in the caffeine‐treated group may reflect caffeine‐induced lipolysis. Although caffeine would be expected to attenuate obesity‐induced elevations in asprosin, the observed increase in both caffeine‐treated groups may be attributed to factors such as animal species, age, degree of obesity, and other experimental conditions. Asprosin, which exerts its effects via receptors expressed in organs such as the liver, brain, kidney, and muscle, is considered a multifunctional hormone. Nevertheless, due to the limited understanding of its physiological and pathological roles, further investigations are warranted (Mazur‐Bialy 2021).

In the present study, plasma adropin levels did not differ between groups. Serum adropin levels have been reported to be lower in rodents experimentally induced with high‐fat and high‐carbohydrate diets compared to the control group (Badawy et al. 2020). Studies have also shown an inverse relationship between adropin levels and BMI (Celik et al. 2013; Sayin et al. 2014). In contrast to these studies, a study examining plasma adropin levels in obese Zucker rats reported no significant difference between obese and healthy rats (Hieda et al. 2019). It has been reported that a specific receptor explaining adropin's mechanism of action in the organism has not yet been identified (Rooban et al. 2024). However, one study suggested that adropin exerts its effect via GPR19 (Stein et al. 2016). Adropin regulates pyruvate dehydrogenase kinase 4 through the p44/42 MAPK signaling pathway, which in turn affects the activity of pyruvate dehydrogenase, a key enzyme in energy metabolism. The mechanisms underlying adropin's role in obesity are still under investigation, and it may exert its effects via multiple signaling pathways, including AMPK, PPARγ, and Sirtuin 1 (SIRT1). These signaling pathways are critically important in energy metabolism and demonstrate that adropin exerts a wide range of physiological effects (Rooban et al. 2024). Examination of studies investigating circulating adropin levels reveals that the experimental designs and the macronutrient composition of the diets used vary considerably. Analysis of these studies indicates that circulating adropin levels increase most prominently in diets high in fat and sucrose (Kumar et al. 2008). In the present study, although significant differences were observed in body weight, BMI, and Lee Index among obese, healthy, and caffeine‐treated rat groups, no differences were detected in plasma adropin levels. This finding suggests that adropin is not directly associated with body weight, obesity, BMI, or Lee Index. Furthermore, the literature contains no studies addressing the effect of caffeine on circulating adropin levels. Therefore, this study represents the first investigation of the relationship between caffeine and adropin, demonstrating that caffeine administration does not significantly affect adropin levels.

Several studies have demonstrated that preptin levels are elevated in obesity (El‐Eshmawy and Aal 2015; Ercan et al. 2019). However, other findings have reported no significant correlation between BMI, obesity, and serum preptin concentrations (Özkaya et al. 2022). In the present study, plasma preptin levels did not differ significantly between the control and obesity groups. Importantly, this study is the first to investigate the effect of caffeine on plasma preptin levels in the context of obesity, revealing a statistically significant difference among the caffeine, control, and obesity groups. This suggests that caffeine may modulate circulating preptin concentrations. Considering that preptin is secreted in response to blood glucose levels and exerts insulin‐mimetic effects, its elevation is expected primarily in diabetes rather than obesity, as also supported by Yang et al. (2009). In line with this, caffeine administration has previously been shown to reduce blood glucose levels in diabetic rats (Urzúa et al. 2012), and in the present study, caffeine‐treated rats exhibited lower preptin levels. These findings indicate that caffeine may lower preptin levels indirectly by reducing blood glucose. Moreover, obesity‐induced insulin resistance is known to promote compensatory hyperinsulinemia in pancreatic β‐cells (Özkaya et al. 2022). The absence of a significant difference in plasma preptin levels between the control and obesity groups in this study may therefore be attributed to the fact that the obese rats had not yet developed pronounced insulin resistance.

In this study, plasma visfatin levels were found to be higher in obese rats compared to the control group. Moreover, this represents the first study to investigate the effect of caffeine use on visfatin levels in obesity. In the literature, obesity studies in experimental animals have reported that serum visfatin levels did not differ between groups (Pérez‐Echarri et al. 2009), whereas other studies have shown increased levels in the obesity group (Abdel‐Fadeil et al. 2019; Gao et al. 2014). In both human and animal experiments investigating the effects of other polyphenols (quercetin, resveratrol, grape polyphenol concentrate, extra virgin olive oil, walnut flour extract) on visfatin levels, it has been reported that polyphenol administration leads to a reduction in visfatin levels (He et al. 2022; Santangelo et al. 2016). Visfatin, an adipokine secreted from visceral adipose tissue, is thought to be elevated in obesity due to the expansion of this tissue. It is hypothesized that caffeine, through its lipolytic effect, may reduce visfatin levels by decreasing adipose tissue. Indeed, the finding that visfatin levels in the obesity + caffeine group were significantly lower compared to the obesity group supports this hypothesis.

In this study, the observed reduction in hepatic β‐catenin levels in obesity supports the contradictory findings reported in the literature regarding different tissues and model systems. Indeed, some experimental animal studies have reported increased β‐catenin activity in the kidney and intestinal mucosa in obesity (Mao et al. 2013; Su et al. 2021), whereas others have reported decreased activity in kidney and liver tissues (Liu et al. 2015; Zhou et al. 2014). Consistent with previous studies on caffeine (Song et al. 2023; Wang et al. 2008) and findings on polyphenols (Luo et al. 2022), no statistically significant differences in hepatic β‐catenin levels were observed between the control and caffeine‐treated groups in the present study. Given the pivotal role of adipose tissue formation in the initiation and progression of obesity, further in‐depth investigations are warranted. The Wnt signaling pathway is a central regulator in the differentiation of mesenchymal stem cells into mature adipocytes (Farmer 2005). Comprehensive mechanistic studies are required to elucidate the precise contributions of this pathway to obesity development and its associated metabolic disturbances.

Studies have reported decreased p38 MAPK levels in the liver tissue of obese mice. In rodents, XBP‐1 is activated via Thr48 and Ser61 phosphorylation following feeding. Inhibition of p38 MAPK prevents the nuclear translocation of XBP‐1 without altering total protein levels; however, this mechanism is impaired in obesity. Although feeding normally increases p38 MAPK, obese rodents show reduced signaling due to pathway resistance, despite elevated inflammation (Lee et al. 2011). Supporting this, MKP‐1 deficiency confers resistance to diet‐induced obesity (Lawan et al. 2018). However, findings regarding p38 MAPK in obesity are inconsistent: in one experimental animal study, an increase was reported (Fang et al. 2019), whereas another experimental animal study reported no change (Kang et al. 2017). The regulatory role of p38 MAPK in adipogenesis is complex and may be species‐specific (Wu et al. 2022). In this study, liver p38 MAPK levels were found to be lower in diet‐induced obese rats compared to controls. These discrepancies indicate that further research is needed to clarify the role of stress‐sensitive MAPKs, particularly p38, in metabolic disorders.

Recent studies have demonstrated that caffeine may reduce liver p38 MAPK levels both in rats (Vargas‐Pozada et al. 2022) and in cell culture models (Ontawong et al. 2023), whereas another cell culture study reported increased activation via MKP‐1 inhibition (Choi et al. 2013). In the present study, p38 MAPK levels were significantly higher in the obesity + caffeine group compared to the obesity group, with the highest levels observed in the caffeine‐only group. Caffeine is thought to activate p38 MAPK by increasing intracellular Ca^2+^ and reactive oxygen species (ROS) through inhibition of MKP‐1 (Liu et al. 2013). These findings provide further insight into the complex interactions between caffeine, stress‐sensitive signaling pathways, and obesity‐related metabolic regulation.

## 5 Conclusion

These findings demonstrate that obesity effectively develops in rats subjected to a cafeteria diet, as evidenced by increases in body weight, BMI, Lee Index, serum glucose, triglycerides, total cholesterol, LDL, and VLDL cholesterol levels, decreases in HDL cholesterol, elevated plasma asprosin and visfatin levels, reductions in liver β‐catenin and p38 MAPK protein levels, and histopathological changes in liver tissue. Caffeine administration mitigated body weight gain, improved lipid profiles, modulated plasma levels of certain adipokines (asprosin, preptin, and visfatin), reduced liver AST and ALT activities, and alleviated histopathological liver alterations. These results indicate that caffeine may exert beneficial effects on multiple metabolic parameters and liver structure during obesity development, although its effects on β‐catenin and p38 MAPK require further investigation.

## Author Contributions

**Lale Baser:** conceptualization (equal), data curation (equal), formal analysis (equal), funding acquisition (equal), investigation (equal), methodology (equal), project administration (equal), resources (equal), software (equal), supervision (equal), validation (equal), visualization (equal), writing – original draft (equal), writing – review and editing (equal). **Emine Atakisi:** conceptualization (equal), data curation (equal), project administration (equal), software (equal), visualization (equal), writing – original draft (equal), writing – review and editing (equal).

## Conflicts of Interest

The authors declare no conflicts of interest.

## Data Availability

The data that support the findings of this study are available on request from the corresponding author. The data are not publicly available due to privacy or ethical restrictions.
